# Supplementary material for: Distinct H3K27me3 and H3K27ac Modifications in Neural Tube Defects Induced by Benzo[a]pyrene
Source: Brain Sci. 2023 Feb 15;13(2):334. doi: 10.3390/brainsci13020334 (PMC9954656; doi:10.3390/brainsci13020334)
Supplement: Supplementary file 1 [file brainsci-13-00334-s001.zip › Additional file S1.pdf]

Supplementary Table S1 Embryotoxicity of BaP in ICR mice.

Group	Litters, n	Embryos, n	Death, n(%)	Resorption, n(%)	Grwoth retardation, n(%)	Cephalic NTDs, n(%)	Other malformations, n(%)
Corn oil	15	180	2 (1.1)	3 (1.7)	3 (1.7)	4 (2.2)	3 (1.7)
BaP-treatment	35	475	22 (4.6)	62 (13.1)	77 (16.2)	78 (16.4)	172 (36.2)

Supplementary Table S2 Sequences of primers for real-time PCR.

Primer	Forward sequence	Reverse sequence
Myl2	CAGATGTTCGCAGCCTTTCC	TACAGTGGATGAGGGCAACTC
Csrp3	GGACAAGAGTCCCCTTACTCAG	CCGTCTTTTCACAGGCTCCA
Grm2	TCAATGCCGTGTATGCCATG	TGTAGCGGCCAATACCATCT
Gapdh	CAGTGAGAAAGTCGGAGTCA	GACACCCATCACAAACATGG

Supplementary Table S3 Descriptive summary of data generated by CUT&Tag for H3K27me3.

Sample ID	Raw Reads	Clean Reads	Clean Ratio (%)	Mapped Reads	Mapping Ratio (%)
Control1	26817910	25725480	95.93%	24808959	89.88%
Control2	22774371	21504001	94.42%	20857323	89.84%
Control3	27730821	26572550	95.82%	25375810	91.96%
BaP-nonNTD1	34304441	32913865	95.95%	32808250	88.98%
BaP-nonNTD2	23047714	22060373	95.72%	21523050	88.22%
BaP-nonNTD3	20219027	19650216	97.19%	19296121	90.31%
BaP-NTD1	29402338	28268280	96.14%	28148111	89.17%
BaP-NTD2	26186193	25141059	96.01%	25004387	87.99%
BaP-NTD3	23213007	22079696	95.12%	21208952	86.87%

Supplementary Table S4 Descriptive summary of data generated by CUT&Tag for H3K27ac.

Sample ID	Raw Reads	Clean Reads	Clean Ratio (%)	Mapped Reads	Mapping Ratio (%)
Control1	26162957	25612134	97.89%	25406736	89.88%
Control2	23957159	23051848	96.22%	22985619	89.84%
Control3	24860346	24031492	96.67%	23787717	91.96%
BaP-nonNTD1	21024432	20232213	96.23%	19896430	88.98%
BaP-nonNTD2	21594953	20793733	96.29%	20424862	88.22%
BaP-nonNTD3	25151970	24605502	97.83%	24395161	90.31%
BaP-NTD1	24443131	23863840	97.63%	23742604	89.17%
BaP-NTD2	23937398	22557980	94.24%	22499179	87.99%
BaP-NTD3	22866977	22075139	96.54%	21889674	86.87%

Supplementary Table S5 Peak widths of H3K27me3 and H3K27ac in E9.5 mouse embryos with and without neural tube defects.

	Control	BaP-nonNTD	BaP-NTD
H3K27me3			
Mean ± SD	289.76 ± 175.75	298.56 ± 208.33	344.44 ± 265.29
P-value		<0.001*	0<0.001*

H3K27ac			
Mean ± SD	378.90 ± 322.39	300.52 ± 254.52	444.30 ± 369.59
P-value		<0.001*	0<0.001*

NTD, neural tube defects; SD, standard deviation; *, compared with control group.

Supplementary Table S18 Descriptive summary of data generated by RNA-seq.

Sample ID	Raw Reads	Clean Reads	Clean Ratio (%)	Mapped Reads	Mapping Ratio (%)
Control1	22720614	22247446	97.92%	19996004	89.88%
Control2	27926555	27342431	97.91%	24564440	89.84%
Control3	23647287	23136116	97.84%	21275972	91.96%
BaP-nonNTD1	23610109	23129880	97.97%	20580967	88.98%
BaP-nonNTD2	20002335	19590948	97.94%	17283134	88.22%
BaP-nonNTD3	24149312	23611317	97.77%	21323380	90.31%
BaP-NTD1	20854309	20393130	97.79%	18184554	89.17%
BaP-NTD2	25403525	24887109	97.97%	21898167	87.99%
BaP-NTD3	23062798	22547272	97.76%	19586815	86.87%
