# Supplementary material for: Distinct H3K27me3 and H3K27ac Modifications in Neural Tube Defects Induced by Benzo[a]pyrene
Source: Brain Sci. 2023 Feb 15;13(2):334. doi: 10.3390/brainsci13020334 (PMC9954656; doi:10.3390/brainsci13020334)
Supplement: Supplementary file 1 [file brainsci-13-00334-s001.zip › Additional file S4.pdf]

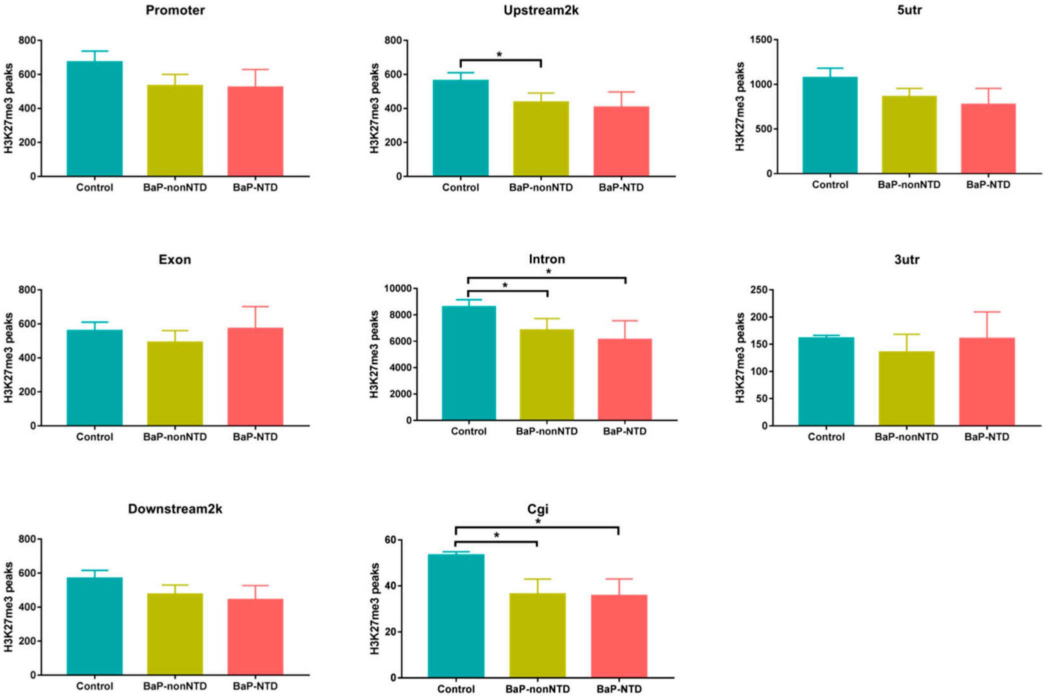

Supplementary figure S1 Genome-wide peak distribution of H3K27me3 in E9.5 embryos with and without neural tube defects. *, *p*-value <0.05, compared to control group.

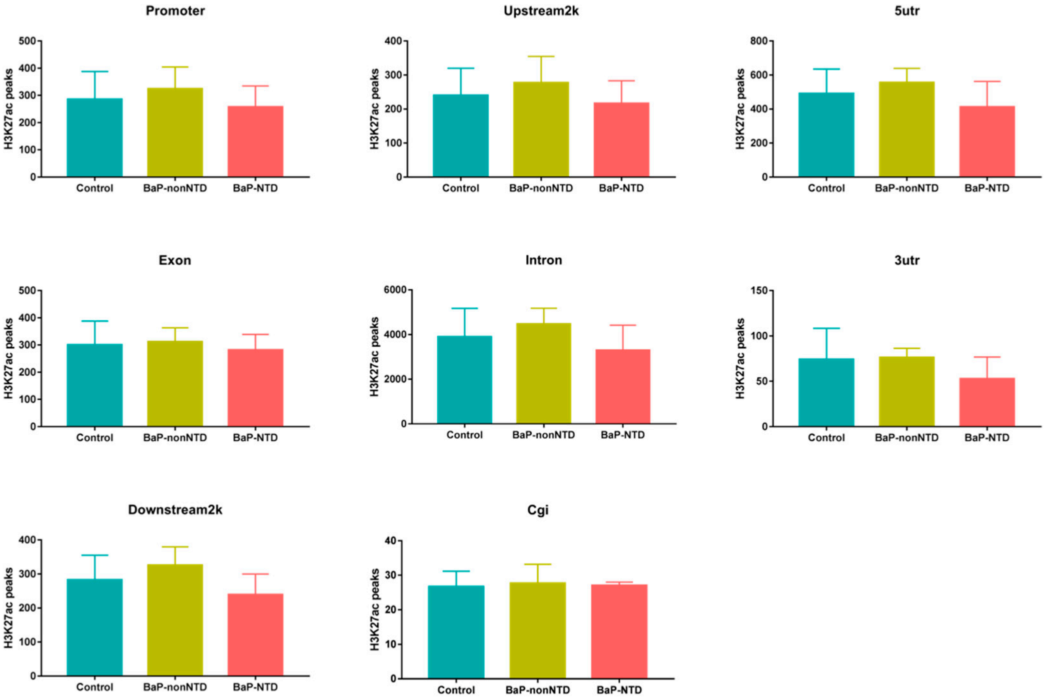

Supplementary figure S2 Genome-wide peak distribution of H3K27ac in E9.5 embryos with and without neural tube defects. *, *p*-value <0.05, compared to control group.

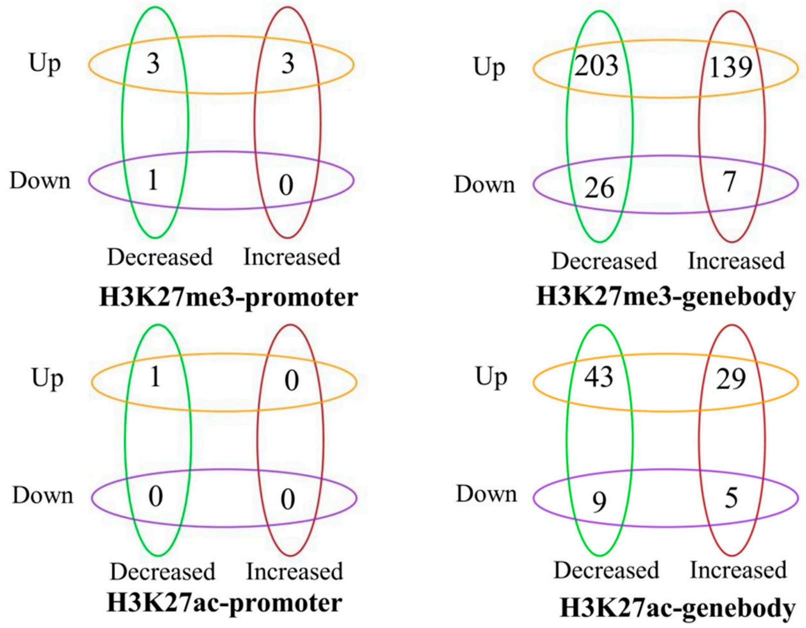

Supplementary figure S3 The relationships between fold change of CUT&Tag signals for H3K27me3/H3K27ac and fold change of gene expressions in BaP-NTD-vs-control comparison.

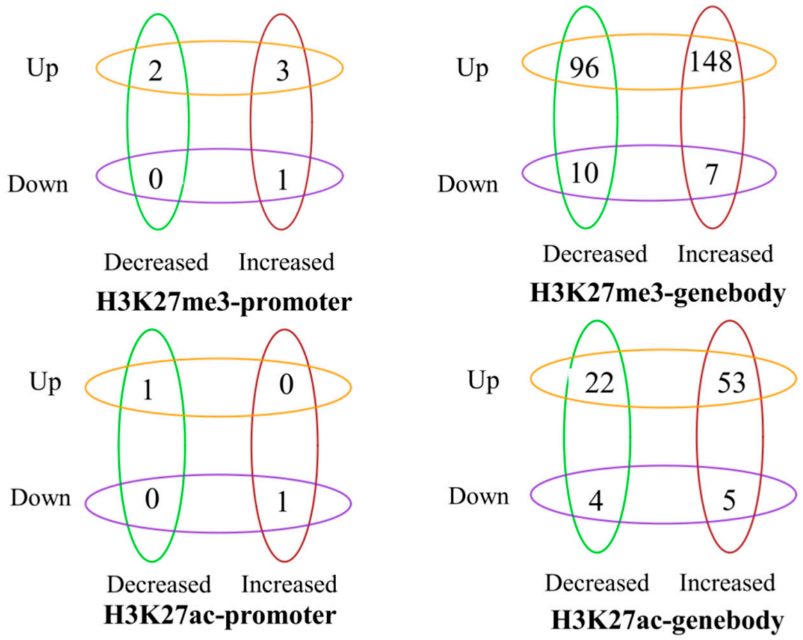

Supplementary figure S4 The relationships between fold change of CUT&Tag signals for H3K27me3/H3K27ac and fold change of gene expressions in BaP-nonNTD-vs-control comparison.

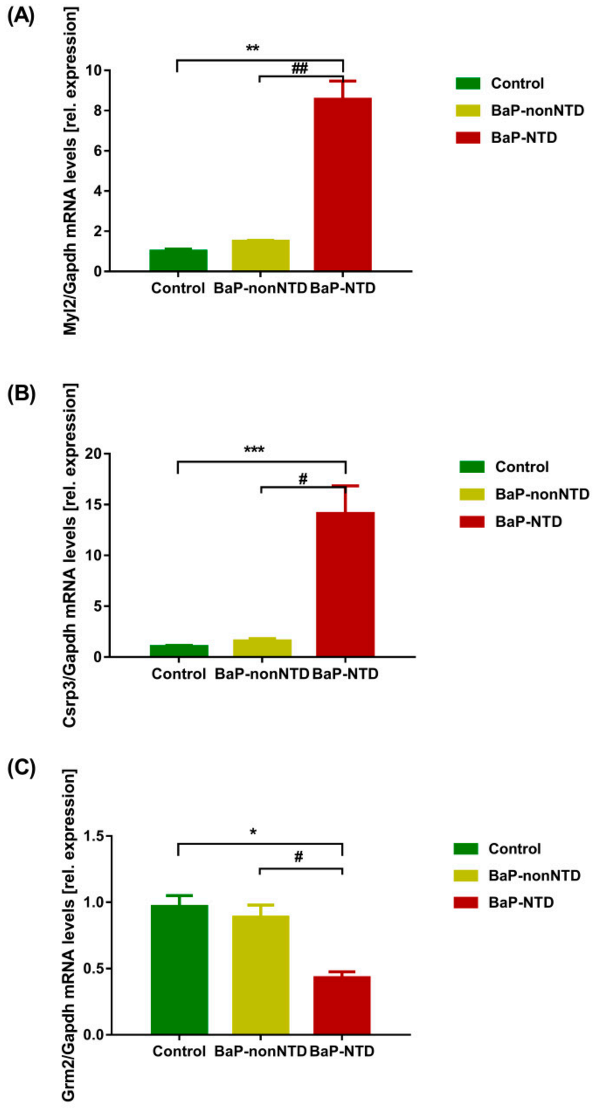

Supplementary figure S5 Relative expression of mRNA of E9.5 embryos E9.5 embryos with and without neural tube defects by real-time PCR. Data for Myl2, Csrp3 and Grm2 were normalized by Gapdh for each sample (mean ± SE; n = 3). *, p < 0.05 or **, p < 0.01 or ***, p < 0.001 vs control; $^{\#}$, p < 0.05 or $^{\#\#}$, p < 0.01 or $^{\#\#\#}$, p < 0.001 vs BaP-nonNTD.
